# Research Progress of Electrically Conductive Asphalt Concrete Deicing and Snowmelt Technology: Material Development and Application Progress

**DOI:** 10.3390/s26061831

**Published:** 2026-03-13

**Authors:** Dong Liu, Jingnan Zhao, Mingli Lu, Zilong Wang, Jigun He

**Affiliations:** 1 Guangxi Xinfazhan Communication Group Co., Ltd., Nanning 530004, China; ldong11@163.com; 2 School of Civil Engineering and Architecture, Guangxi University, Nanning 530004, China; 2410391089@st.gxu.edu.cn; 3 Guangxi Transportation Science and Technology Group Co., Ltd., Nanning 530004, China; zilongww@gmail.com; 4 Guangxi Transportation Construction Engineering Group Co., Ltd., Nanning 530004, China; 18178138218@163.com

**Keywords:** ECAC, de-icing and snow melting, conductive fillers, Joule heating effect, intelligent transportation systems

## Abstract

Snow accumulation and ice formation can significantly reduce pavement friction, posing a serious threat to traffic safety during winter. Traditional snow-removal methods, including mechanical removal, chemical de-icing agents, and heated pavement systems, suffer from several limitations such as low efficiency, environmental impacts, and high operational costs. Electrically conductive asphalt concrete (ECAC) has therefore emerged as a promising active snow-melting technology. When an electric current passes through the conductive network formed within the asphalt mixture, heat is generated through the Joule heating effect. After incorporating conductive fillers, the electrical resistivity of ECAC mixtures can be reduced from approximately 10^6^–10^8^ Ω·cm for conventional asphalt mixtures to about 10^−1^–10^2^ Ω·cm. Under an applied voltage typically ranging from 30 to 60 V, ECAC pavements can increase the surface temperature by 10–30 °C within 10–30 min, thereby enabling rapid snow melting and ice removal. Meanwhile, an optimized conductive network can maintain sufficient mechanical performance, with dynamic stability generally exceeding 3000 cycles/mm. When the conductive filler content is reasonably controlled, only a limited reduction in fatigue resistance is observed. This paper presents a comprehensive review of electrically conductive asphalt concrete technologies for snow-melting pavements. The background, underlying mechanisms, material development, system configuration, and field applications of ECAC are systematically summarized. Finally, the current challenges are discussed, including the stability of conductive networks, the trade-off between electrical conductivity and pavement performance, and electrical safety. Future research directions focusing on material optimization, intelligent power control, and long-term field performance evaluation are proposed to support the practical application of ECAC pavements in sustainable winter road maintenance.

## 1. Introduction

### 1.1. Background and Importance of Deicing and Snowmelt Technology

#### 1.1.1. Impact of Ice and Snow on Traffic Safety and Economy

Accumulated snow and ice significantly impair transportation systems, posing severe safety risks and causing substantial economic disruptions to logistics and supply chains. Adverse winter weather drastically elevates the risk of traffic accidents. According to the Federal Highway Administration (FHWA), approximately 22% of vehicle collisions occur on slippery surfaces, with 17% specifically attributed to snow or ice conditions [1]. Notably, freezing precipitation poses a higher traffic safety risk than snowfall [2]. Beyond safety concerns, weather-related delays impose a heavy economic burden. A 2012 FHWA study estimated that such delays cost the U.S. trucking industry between $8 billion and $9 billion annually. In specific highway corridors, weather-induced speed reductions can result in $3.8 million in annual losses for the freight sector [3]. Furthermore, icy road conditions hinder vehicle navigation, disrupting the transportation of raw materials and finished products [4]. A prime example is the 2021 winter storm “Hurricane Uri”, which exposed supply chain vulnerabilities and caused severe shortages of critical goods, including medical equipment [5].

#### 1.1.2. Limitations and Challenges of Traditional Ice Removal and Snow Melt Methods

Traditional de-icing and snow-removal methods mainly rely on chemical salts and mechanical clearing [6]. Despite their widespread use, these approaches pose considerable environmental and ecological risks. Chloride-based de-icing agents, particularly sodium chloride (NaCl), exhibit high mobility and poor biodegradability, leading to chloride accumulation in soil, surface water, groundwater, and roadside vegetation [7,8]. Chloride-rich runoff degrades soil and water quality, threatens aquatic ecosystems and drinking water safety, and may induce secondary salinization [8]. Elevated salinity levels can exceed chronic toxicity thresholds for aquatic organisms, impairing physiological functions, reproduction, and immune responses [9]. Moreover, saline irrigation adversely affects agricultural productivity by increasing osmotic stress and ion toxicity, thereby inhibiting plant growth and altering fruit morphology [10].

Salt application adversely affects infrastructure durability by inducing salt scaling, which compromises pavement surfaces and structural components. Owing to the hygroscopic nature of de-icing salts, corrosion may occur even under low relative humidity conditions [11]. Experimental studies demonstrate that saline environments accelerate asphalt pavement degradation. After 15 wet–dry and freeze–thaw cycles in 5% and 10% NaCl solutions, the stiffness modulus of asphalt–limestone mixtures increased by 20.2% and 38.2%, respectively [9]. This behavior is attributed to the weaker adsorption between NaCl and asphalt molecules compared with binder–binder interactions, resulting in reduced cohesive strength and accelerated stripping [12]. Additionally, NaCl exposure decreases asphalt ductility by disrupting intermolecular bonding and altering the compositional balance of asphalt fractions [13]. Mechanical damage is further intensified by improper snowplow height settings, leading to sealant delamination and aggravated pavement deterioration [14].

Furthermore, conventional methods suffer from limited operational efficiency. These approaches are energy-intensive, labor-intensive, and often lack broad applicability [7]. Most passive methods only respond after ice formation, making them less effective than active de-icing strategies, while repeated salt applications consume significant fuel and materials [15].

### 1.2. Demand for New Deicing Technologies in Intelligent Transportation and Sustainable Development

Driven by global initiatives for smart cities, Intelligent Transportation Systems (ITS), and Sustainable Development Goals (SDGs), the development of efficient, eco-friendly, and energy-saving de-icing technologies has become a critical priority. ITS focuses on systematic optimization to alleviate traffic congestion and mitigate pollutant emissions, fostering a synergy between environmental preservation and economic growth [16]. Furthermore, the widespread adoption of Advanced Driver Assistance Systems (ADAS) and Autonomous Vehicles (AVs) imposes more stringent requirements on pavement surface clarity [17], but also generate glare and splashes that can trigger system malfunctions, directly compromising vehicle perception and control [18]. To overcome path recognition challenges in complex winter environments, research is accelerating on both in-vehicle anti-icing systems and active de-icing infrastructure [19].

The Sustainable Development Goals (SDGs) have driven the development of innovative and sustainable de-icing technologies. Within sustainable transportation systems, reducing carbon emissions and environmental pollution has become a key objective [20]. Accordingly, modern de-icing strategies focus on improving road safety while minimizing ecological impacts by promoting active anti-icing approaches, reducing salt usage, and developing alternative de-icing agents [21]. Under policy support and technological advances, bio-based modifiers, low-impact salts, and organic deicers have emerged as major research directions [22,23,24]. For example, de-icing agents incorporating starfish extracts have demonstrated effective performance with substantially reduced corrosivity [25]. From a life-cycle perspective, sustainable road maintenance prioritizes efficient resource allocation and cost control. In this context, anti-icing techniques outperform conventional reactive methods due to higher efficiency and lower material demand. Advanced systems such as GPS-controlled precision spreaders, weather-responsive technologies, and remote monitoring platforms further optimize operations [26,27].

### 1.3. Overview and Advantages of ECAC Deicing Technology

To address the growing demands of intelligent transportation systems and sustainable development, ECAC has emerged as a promising active de-icing technology with distinct advantages over conventional methods. By embedding electrodes and sensors within the asphalt surface layer, snow and ice are removed through Joule heating, while real-time sensing enables adaptive control of thermal efficiency. Given the inherently low electrical conductivity and thermal transmittance of conventional asphalt, conductive fillers are commonly introduced to enhance electrothermal performance. For example, the CREATES team at Rowan University incorporated 30% graphite and 1% carbon fiber (by volume) into the asphalt binder, reducing the mixture resistivity to 1–2 Ω·m [14]. In practical applications, the conductive layer thickness is typically controlled within 15–25 mm to balance material cost and energy consumption [28].

Compared with conventional de-icing methods, ECAC provides several distinct advantages [29]. By eliminating the need for snowplows and chemical de-icing salts, ECAC reduces mechanical pavement damage and avoids the acceleration of freeze–thaw deterioration caused by salt crystallization [14]. This technology is particularly suitable for safety-critical infrastructure, such as airport pavements, bridges, and high-traffic highways, where continuous serviceability and a high level of traffic safety are essential [30]. Moreover, the complete elimination of de-icing salts directly mitigates the environmental impacts associated with chemical methods and supports the objectives of the Sustainable Development Goals [19]. Unlike passive conventional approaches, ECAC enables active and automated de-icing, improving operational efficiency and reducing labor demand during winter conditions [9]. A comparative summary of the two strategies is presented in Table 1.

### 1.4. Research Objectives and Structure of This Paper

This paper presents a comprehensive review of ECAC de-icing technology to clarify its research progress and application trajectory. The review first outlines the technical background, emphasizing the impacts of snow and ice on transportation systems and the limitations of conventional de-icing methods. It then introduces the fundamentals of ECAC and the overall research framework. Key technical aspects—including conductive mechanisms, critical fillers, system configurations, and field applications—are systematically examined. Subsequently, the economic and environmental benefits are assessed, followed by a discussion of current challenges and emerging research trends. Finally, future research directions are proposed to support technological optimization, engineering implementation, and the development of sustainable transportation infrastructure.

## 2. Traditional De-Icing and Snow-Melting Technologies

### 2.1. Mechanical Snow Removal Methods

**Figure 1:**
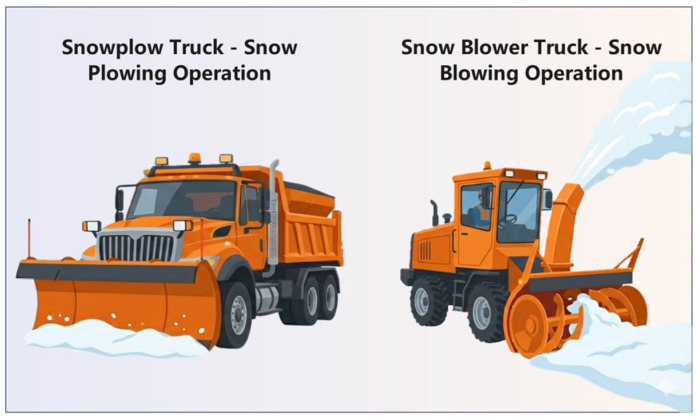
Mechanical snow removal, snowplow truck and snow blower truck.

Mechanical snow removal is a conventional winter maintenance approach based on the physical clearance of accumulated snow [31]. Common equipment includes snowplows and snow blowers: plows push snow toward the roadside, whereas blowers remove and eject snow from the pavement at high velocity [32]. Snow accumulation represents a major logistical and economic challenge [33]. As shown in Figure 1, two common types of mechanical snow removal are illustrated, snowplow truck and snow blower truck.

The primary advantage of mechanical snow removal is its direct and efficient physical clearance capability. When combined with pre-applied anti-icing measures, mechanical operations can substantially reduce the demand for chemical deicers and improve overall removal efficiency [32]. However, mechanical removal also has inherent limitations. The first is the slow pace of equipment renewal and sluggish efficiency improvement [34,35]. The second is insufficient power output. It fails to maintain an effective clearing speed of 32.19–48.28 km/h during moderate to heavy snowfall. Under the conditions of handling wet snow with a depth of 7 inches and a density of 432.5 kg/m^3^, an operational cross-section of 8 square feet, achieving a snow casting volume of 261 tons per minute and a casting distance of 45 feet, maintaining a speed of 40.23 km/h typically requires more than 400 horsepower [33]. Additionally, the majority of snow-removal vehicles rely on fossil fuels, generating substantial greenhouse gas and pollutant emissions [36].

### 2.2. Chemical De-Icing Methods

#### 2.2.1. Chloride-Based De-Icing Agents

Chloride-based agents are the most widely used and cost-effective chemical deicers, including sodium chloride (rock salt), calcium chloride, and magnesium chloride [37]. In addition to lowering the freezing point, calcium and magnesium chlorides release heat through exothermic reactions, accelerating ice melting [38]. Liquid deicers are more effective than solid deicers in preventing the bonding between ice layers and the pavement surface [39]. For light snowfall of 5–7.5 cm, these chemical solutions remain an efficient and economical maintenance strategy [31].

**Figure 2:**
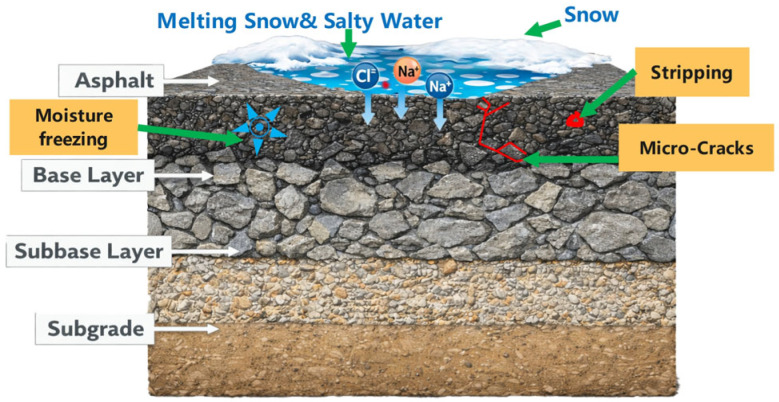
Schematic illustration of moisture-induced damage mechanisms in asphalt pavements under winter deicing conditions, including moisture freezing, stripping, and micro-crack formation.

Chloride salts can lead to both physical and chemical deterioration of concrete [40]: physical damage includes scaling, salt crystallization damage, and the exacerbation of freeze–thaw cycles [41]. Chemical damage, on the other hand, manifests as the leaching of calcium hydroxide, the formation of oxychlorides, and an increase in permeability. Figure 2 illustrates the mechanism of chloride damage to asphalt pavements.

Chloride ions can penetrate concrete and disrupt the passive layer of steel reinforcement, promoting corrosion. The resulting corrosion products expand, generating internal tensile stresses, cracking, and spalling, which shorten structural service life [42]. Magnesium chloride (MgCl_2_) can induce severe cracking even without freeze–thaw cycles, due to the formation of expansive phases such as magnesium oxychloride (e.g., 5Mg(OH)_2_·MgCl_2_·8H_2_O) and magnesium–silicate–hydrate (M–S–H), as well as Friedel’s salt (3CaO·Al_2_O_3_·CaCl_2_·10H_2_O) formed indirectly from liberated calcium ions; these phases collectively replace the binding calcium–silicate–hydrate (C–S–H) [35]. Sodium chloride (NaCl) further exacerbates freeze–thaw damage by increasing osmotic pressure and triggering non-ideal phase transitions during freezing.

#### 2.2.2. Non-Chlorinated Salt De-Icing Agents

To mitigate the detrimental effects of chloride-based de-icing agents, various non-chlorinated alternatives have been developed and deployed. These non-chloride de-icers are primarily categorized into several functional groups, as summarized in Table 2.

Non-chloride de-icing agents differ from chloride-based salts in performance and environmental impact. Formates and glycols are effective within −28.9 °C to −9.4 °C, while urea peaks between −9.4 °C and 0 °C [43]. Potassium acetate melts ice more efficiently than sodium chloride, and potassium succinate can penetrate ice at temperatures as low as −20 °C [48]. However, non-chloride deicers also pose environmental concerns. Their decomposition can lead to a high biochemical oxygen demand (BOD), which negatively impacts aquatic organisms [49,50]. In addition to depleting dissolved oxygen, they can also cause toxic contamination of water sources [51]. This trade-off between long-term cumulative pollution and short-term oxygen-depleting effects requires evaluation based on specific environmental conditions.

Non-chloride de-icing agents are generally significantly more expensive than their chloride-based counterparts. For example, calcium chloride costs 40% or more than sodium chloride [52]. Acetate prices typically exceed $2000 per ton, far surpassing the cost of sodium chloride [53]. Similarly, formates and glycol-based products (glycerol/ethylene glycol) command relatively high market prices. Table 3 presents a comparative analysis of common de-icing agents, focusing on their performance, cost-efficiency, and environmental impact:

### 2.3. Thermal-Based De-Icing Techniques

#### 2.3.1. Geothermal System

**Figure 3:**
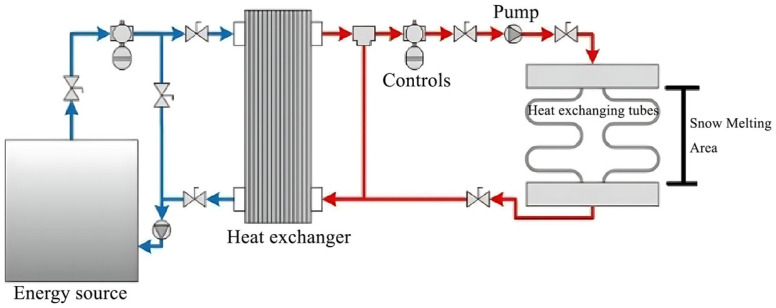
Geothermal system for road surfaces [58].

Geothermal de-icing systems primarily utilize ground source heat pumps (GSHP) or hydronically heated pavement (HAP) systems to transfer heat to the pavement surface [54,55]. Ground source heat pumps extract shallow geothermal energy through horizontal or vertical pipes buried underground. Their coefficient of performance (COP) typically ranges from 3 to 6, meaning that for every 1 kW of electrical energy consumed, 3 to 6 kW of thermal energy is generated [56]. Geothermal energy is consistently available and largely unaffected by weather fluctuations. Peak heat pump output can reach 59.4 °C, with daily average heating above 25.2 °C [57]. Operational efficiency of hydraulic geothermal systems depends on factors such as effluent temperature, surrounding soil properties, and allowable reinjection temperatures. Figure 3 illustrates a system that utilizes geothermal energy to melt snow on road surfaces. Heat is transferred to the snow-melting zone via underground heat exchangers, pumps, and a control system [58].

#### 2.3.2. Electric Heating System

**Figure 4:**
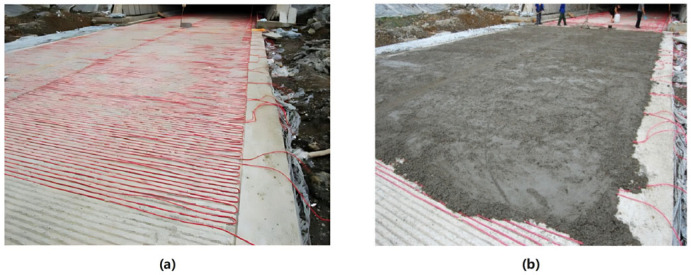
On-site construction of heated pavement: (**a**) Heating wire layout; (**b**) Pavement paving [61].

Electric heating systems typically employ electrically conductive concrete (ECON), which utilizes electrodes to convert electrical energy into Joule heat for snow melting and de-icing [59]. Ordinary concrete is an insulator and requires the incorporation of conductive fillers such as carbon fibers or steel fibers to form a conductive network. Some studies recommend a carbon fiber content of 0.40 vol.% [60]. The arrangement of shallow electrodes should be optimized as much as possible to enhance the surface thermal response capability and improve energy utilization efficiency [60]. Figure 4 illustrates the construction process of an electrically heated pavement: (a) heating cables are evenly laid on the base layer, (b) followed by paving the surface layer to encapsulate the cables, ultimately forming an active snow-melting pavement [61].

Electrically heated pavement systems typically achieve an overall system energy efficiency of ~50% for snow and ice melting, defined as the ratio of latent heat effectively used for phase change to the total electrical energy input [62]. Precise control of ECON electrical resistance is essential: excessive resistance reduces power output, while insufficient resistance increases current draw and energy consumption, potentially damaging the system. For carbon fiber-reinforced ECON, the percolation threshold ranges from 0.50–0.75 vol.% in lab samples, and around 0.35 vol.% in industrial production [60,63].

Electrically heated pavement systems aim to reduce long-term operational costs and maintain year-round traffic flow. Electrical safety is critical, particularly in humid environments where users may be exposed. Human skin resistance ranges from 1 kΩ to 100 kΩ depending on humidity and contact area, while the system resistance is much lower [64].

### 2.4. Problems and Development Bottlenecks of Traditional Technology

Conventional winter maintenance—mechanical removal, chemical de-icing, and thermal melting—faces systemic limitations that hinder long-term sustainability. Key challenges include high life-cycle costs, environmental impacts, accelerated pavement deterioration, limited operational efficiency, and seasonal applicability. Additional issues such as high labor intensity, safety risks, and fragmented technological approaches highlight the need for advanced, multi-functional, and self-maintaining pavement solutions.

These challenges create systemic bottlenecks in traditional de-icing and snow-melting methods, limiting their ability to meet modern requirements for road safety, environmental protection, and economic sustainability. Innovative solutions are needed to overcome these limitations and improve life-cycle cost-effectiveness. ECAC de-icing technology offers a promising paradigm shift by combining structural integrity with active self-heating, addressing the economic, environmental, and operational shortcomings of conventional approaches and enabling a more sustainable, efficient, and resilient winter road maintenance strategy.

## 3. Principle and Classification of ECAC Deicing and Snowmelt Technology

### 3.1. Conductive Mechanism of ECAC

#### 3.1.1. Percolation Theory

**Figure 5:**
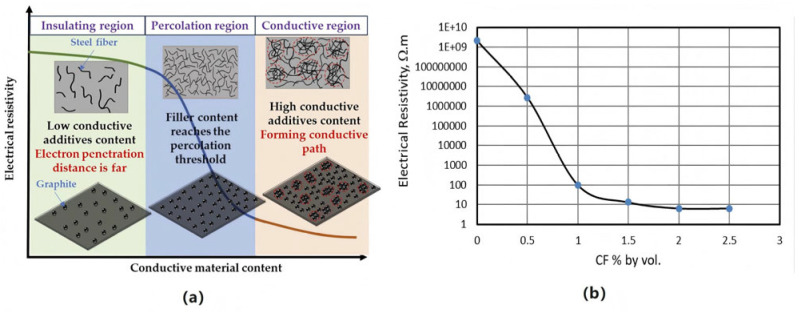
Percolation behavior of electrical resistivity in ECAC: (**a**) Percolation Threshold Mechanism of ECAC; (**b**) Experimental electrical resistivity versus carbon fiber (CF) volume fraction [67,68].

The electrical conductivity of ECAC depends primarily on the structure of the conductive network rather than filler concentration alone. Percolation theory explains this behavior: as conductive filler content reaches a critical volume fraction, resistivity drops sharply, marking the formation of continuous conductive pathways, as shown in Figure 5a [65,66]. For example, increasing carbon fiber content from 0.5 vol.% to 1 vol%. can reduce resistivity by over four orders of magnitude [67], as shown in Figure 5b.

The percolation threshold occurs over a narrow filler concentration range, where small variations can cause order-of-magnitude changes in resistivity. This sensitivity complicates precise tuning of electrical properties and poses challenges for consistent quality control of ECAC in multifunctional applications.

#### 3.1.2. Formation Mechanism of Conductive Network

The bulk conductivity of ECAC depends not only on filler content but primarily on the spatial distribution and topology of the conductive network. Network efficiency and robustness are influenced by the morphology (aspect ratio, surface area), chemical composition, and dispersion state of conductive phases within the asphalt matrix [65,69].

**Figure 6:**
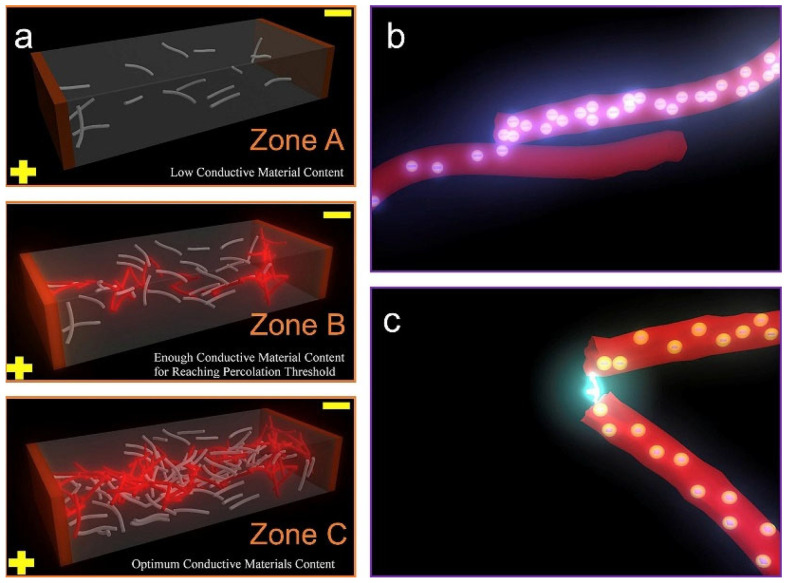
A schematic diagram illustrating the evolution of the conductive network and its primary conduction mechanisms in ECAC: (**a**) Relationship between resistivity and additive content: Zone A insulating phase; Zone B transitional phase; Zone C excess conductive phase; (**b**) contact conduction; (**c**) tunneling effect [77].

Asphalt mixtures are inherently dielectric materials, characterized by a lack of electrical conductivity and acting as near-perfect insulators [70]. Electrical conductivity of ECAC is achieved through the strategic integration of functional conductive additives. The resulting charge transport behavior is governed by a combination of synergistic mechanisms, primarily including contact conduction, the quantum tunneling effect, field emission, and ionic conduction, as shown in Figure 6 [29,71,72,73].

In ECAC, the tunneling effect allows electrons to cross nanoscale gaps between fillers, while direct contact forms continuous pathways enabling Ohmic conduction [73]. When particles are separated by thin insulating films, thermal vibrations activate tunneling, and high local electric fields induce field emission currents. Conductive additives with smaller diameters or higher aspect ratios enhance these quantum effects [74]. For example, Yan et al. proposed an equivalent circuit model (R1) to describe the charge transport behavior in ECAC systems. Their analysis suggested that at low filler content, electron transport is largely governed by tunneling-assisted conduction due to insufficient direct contact between conductive phases, whereas with increasing filler content and network formation, contact-based conduction becomes progressively more significant [75]. Quantitative analyses in asphalt-based systems under typical DC heating conditions (low voltage < 60 V) indicate that: (1) at low conductive filler contents, electron tunneling dominates; (2) when the filler content reaches the percolation threshold, tunneling and contact conduction jointly dominate; (3) as the filler content continues to increase, contact conduction becomes predominant, while field emission plays only a secondary role at high local electric fields. Ionic conduction remains negligible due to the extremely low ion mobility in the dry asphalt matrix [68,76].

Carbon fiber powder (CFP) is a prevalent conductive filler in asphalt concrete; however, its integration has been reported to compromise the interfacial bonding performance of the asphalt mortar [69]. Despite its widespread use, the precise micromechanisms governing this adhesive degradation remain elusive. To bridge this knowledge gap, researchers have utilized X-ray computed nanotomography (Nano-CT) to characterize the spatial distribution of CFP and its correlation with electrical performance. These advanced characterizations have helped to elucidate a “long-short synergistic” conductive mechanism, where the multiscale interactions between conductive phases optimize the formation of the electron transport network [68].

For granular fillers like graphite, effective conduction requires an inter-particle spacing of ~2 εm; below 0.68 vol.% graphite, conductivity drops sharply [68]. Hybrid systems combining carbon fibers and graphite form more resilient networks: graphite clusters enable short-range transport, while carbon fibers bridge these clusters, creating continuous conductive pathways at lower total filler content [78]. The microstructure of asphalt concrete, particularly air voids, strongly influences electrical performance. Higher porosity disrupts conductive networks, increasing resistivity [79]. Monitoring resistivity variations enables non-destructive structural health assessment and supports proactive pavement maintenance.

### 3.2. Heating Mechanism of ECAC

#### 3.2.1. Joule Heat Effect

The Joule heat effect, also known as resistance heating, is the fundamental principle for snow and ice removal achieved through ECAC [80]. This effect refers to the conversion of electrical energy into thermal energy when current passes through a material with resistance. In ECAC, the current can directly generate heat within the material [81].

According to Joule’s law, the electric current in a material generates sufficient heat to effectively prevent snow accumulation and ice formation on roads. This law states that heat is directly proportional to the material’s resistance, the duration of current flow, and the square of the current, as shown in Formula (1):

*H* = *I*^2^*Rt* (1)

where *H* is heat, *I* is current, *R* is resistance, and *t* is the duration of current flow.

A typical ECAC deicing system features an integrated architecture, primarily comprising: a ECAC mixture layer, resistance cables, power supply, environmental monitoring sensors, and an intelligent control system. These components work in concert to ensure efficient and safe operation under low-temperature conditions [82]. The Joule heat effect serves as the key mechanism in ECAC deicing, with its efficiency directly determining the deicing performance [83]. However, heat generation and transfer are influenced by multiple factors, including material conductivity, thermal conductivity, and external environmental conditions [84].

#### 3.2.2. Temperature Field Distribution and Heat Conduction

Thermal exchange in conductive pavement involves conduction, convection, and radiation [85]. In ECAC, heat arises from ionic conduction in pore solutions and electronic conduction through fiber networks, then distributes via matrix conduction. Convection occurs mainly at pavement–ice/air interfaces, while long-wave radiation dissipates heat to the environment.

**Figure 7:**
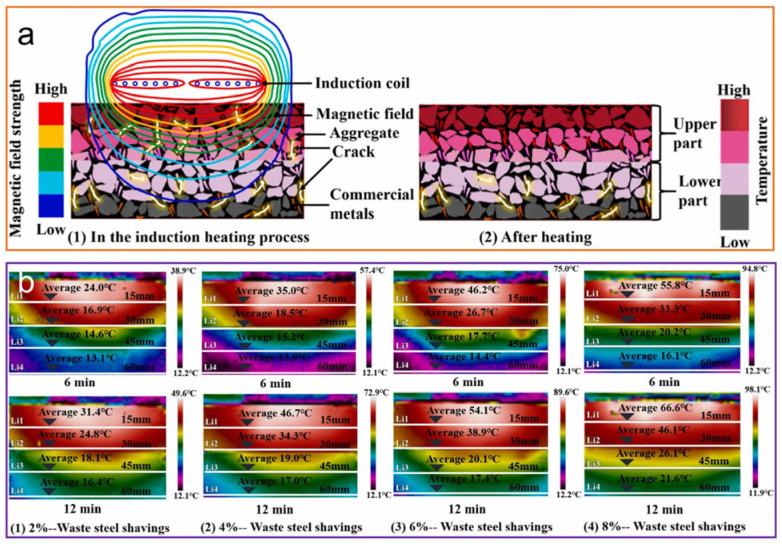
Magnetic field and temperature field distribution of waste steel shavings modified asphalt mixture under induction heating, and internal temperature variation with different dosages and heating durations: (**a**) Induction heating of conventional induction-healing asphalt mixture and (**b**) Temperature distribution field of ECAC containing waste steel chips [86].

**Figure 8:**
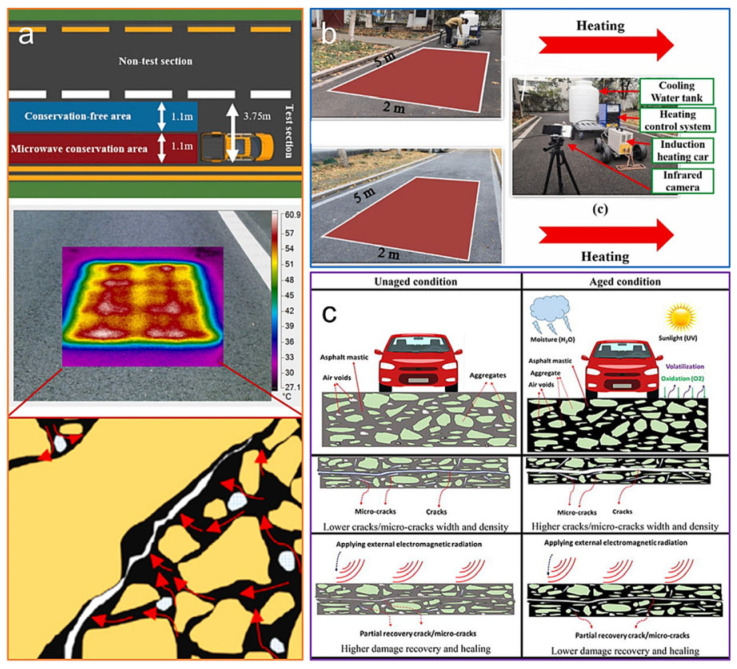
ECAC and its application in self-healing of asphalt pavements: (**a**) Zoning map of the protected area and boundaries of microwave curing zones; (**b**) Dynamic induction heating vehicle and infrared temperature distribution; (**c**) Effect of aging levels on the heating healing capacity of ECAC [77].

Fu et al. [86] addressed the non-uniform temperature gradient distribution in ECAC pavements by employing a layered functional design, incorporating scrap steel chips in the upper layer and steel wool fibers in the lower layer (Figure 7). In a separate approach, Sun et al. [87] evaluated the heating performance of steel fiber-modified ECAC under microwave irradiation, simulating the operational intensity of a 90 kW maintenance vehicle. Their results indicated that the pavement surface heating rate reached an impressive 26 °C during microwave-induced curing (Figure 8a). Furthermore, Fu’s team [86] developed a sustainable ECAC mixture utilizing metallic waste materials for inductive self-healing. In short-duration high-power induction heating experiments aimed at crack repair, a peak surface temperature of 93.5 °C was achieved (Figure 8b). Although this peak temperature is effective for rapid healing, prolonged exposure above 70 °C risks accelerating binder aging through oxidation and volatilization. In routine de-icing applications, operational surface temperatures are therefore controlled to 5–15 °C above freezing using smart sensors and power modulation to ensure efficient snow/ice melting while avoiding long-term aging [88,89]. However, Amani and collaborators [90] noted that the induction heating efficiency tends to attenuate with prolonged service life (Figure 8c).

## 4. Research and Development of Key Materials for ECAC and Performance

### 4.1. Types, Properties of Conductive Fillers and Their Effects on Asphalt Performance

#### 4.1.1. Carbon-Based Conductive Materials

Carbon-based fillers, including graphene, graphite, carbon nanotubes (CNTs), and carbon black [91], are extensively utilized in asphalt modification owing to their superior mechanical, electrical, and thermal properties [92]. Among these, carbon nanotubes (CNTs) represent a prominent class of nanomaterials, characterized by exceptional tensile strength and high thermal and electrical conductivities. In particular, certain single-walled carbon nanotubes (SWCNTs) exhibit remarkable structural integrity and performance [93]. CNTs are generally categorized into two types: single-walled carbon nanotubes (SWCNTs), with diameters ranging from 0.5 to 2.0 nm, and multi-walled carbon nanotubes (MWCNTs), which consist of multiple coaxially nested graphene layers [94]. The extraordinary performance of CNTs originates from their nanoscale architecture, governed by the volume effect, tunneling effect, and size effect, alongside the robust covalent bonding between carbon atoms.

The thermal and electrical performance of asphalt composites is strongly influenced by CNT characteristics, including length, dispersion, and orientation [95]. High-aspect-ratio, well-dispersed CNTs form continuous thermal and electrical pathways, enhancing bulk conductivity and lowering percolation thresholds [96]. Single-walled CNTs (SWCNTs) generally outperform multi-walled CNTs due to their superior atomic-scale structure [94].

Graphene exhibits excellent electrical and thermal conductivity and stability, enhancing asphalt binder performance [97]. At <1.5 wt.% doping, graphene disperses effectively in asphalt [98], but high cost limits large-scale use. Alternatives like multi-layer graphene (MLG) or functionalized graphene nanoplatelets (GNPs) provide a cost-effective compromise with slightly lower intrinsic conductivity [99].

ECAC design requires balancing electrical performance with cost [100]. Graphene integration evaluates both conductivity and economic feasibility. Micron-scale graphite, especially crystalline forms, provides high conductivity, but excessive filler can compromise mechanical integrity. Optimizing graphite crystallinity and particle size achieves percolation at lower dosages while preserving structural performance [65,82].

#### 4.1.2. Metal-Based Conductive Materials

**Figure 9:**
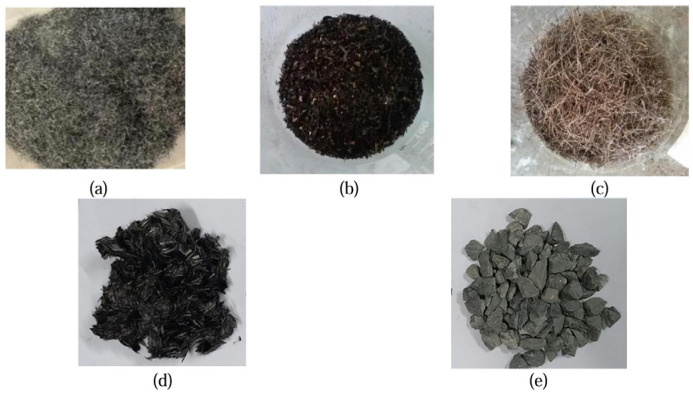
Conductive Fillers Used for the Preparation of ECAC: (**a**) Steel wool; (**b**) Steel chips; (**c**) Steel fibers from waste tires; (**d**) Carbon fibers; (**e**) Iron tailings aggregate [105].

Metal-based fillers, such as steel fibers, aluminum fibers, steel wool, nickel powder, copper slag, and iron tailings, enhance asphalt conductivity [101,102,103]. Micron-scale steel fibers efficiently form robust conductive networks while providing reinforcement, outperforming traditional graphite fillers [104]. Common metal-based conductive materials are shown in Figure 9. These materials offer a sustainable and economical option for heated road surfaces.

One study reported that the critical embedded steel fiber length for maximizing micro-crack bridging and robust conductive network formation is approximately 9.6 mm [29]. In the asphalt matrix, fibers form a 3D reinforcement skeleton, enhancing load transfer and stress distribution. This integration converts “free asphalt” into “structural asphalt”, simultaneously improving electrical conductivity and mechanical properties, including Marshall stability, rutting resistance, ITS, and low-temperature fracture toughness [104,106], supporting high-performance multifunctional ECAC.

Excessive steel fiber content can reduce Marshall stability and increase air voids due to high flexural stiffness, which impedes mixture compaction [107,108]. Incorporating industrial by-products, such as iron tailings (TA) and copper slag (CS), can provide conductivity while improving mechanical performance. For example, iron tailings form interconnected networks, and combining copper slag with carbon fiber (CF) effectively tunes composite resistivity [109,110,111].

#### 4.1.3. Novel Composite Conductive Materials

**Figure 10:**
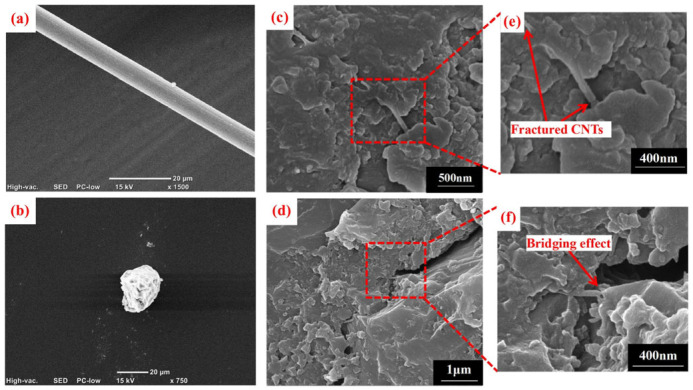
Scanning electron microscopy (SEM) images showing the morphology of carbon nanotubes (CNTs) and their interaction mechanisms in the asphalt mortar matrix: (**a**) Morphology of a single conductive CNT fiber; (**b**) Agglomerated CNT particle; (**c**,**d**) Typical fracture surface morphologies of the CNT-modified asphalt mortar at different magnifications; (**e**) Fractured CNTs embedded in the matrix; (**f**) Bridging effect of CNTs in the asphalt matrix [116,117].

Multifunctional asphalt increasingly relies on hybrid conductive systems. Combining fillers, such as steel fibers and graphite, exploits synergistic effects to optimize electrical resistivity and network robustness [78,112]. In these composites, particulate fillers form local clusters, while elongated fibers bridge them, creating a continuous and resilient conductive network, as shown in Figure 10 [113].

Recent research emphasizes synergistic use of industrial by-products and recycled materials with conductive fillers to balance mechanical performance and sustainability. For example, graphene nanoplatelets (GNPs) with electric arc furnace slag (EAFS) exhibit thermistor behavior for self-sensing applications [114]. Systems combining copper slag (CS) and chopped carbon fiber (CF) enable high-performance ECAC [115]. Hybrid blends of steel slag, graphite, and carbon/steel fibers can reduce volume resistivity below 10 Ω·m, linking waste utilization with functional pavement engineering [103].

#### 4.1.4. Comparative Evaluation of Conductive Fillers

Although it has been reported that various conductive fillers can enhance the performance of ECAC, direct comparisons remain limited due to the differing test conditions across studies. In general, carbon-based fillers exhibit a relatively low percolation threshold and a significant reduction in resistivity, while having a relatively mild impact on mechanical properties. Metallic fillers, on the other hand, provide a stable conductive network and crack-bridging effects that improve fatigue life, but they typically require higher dosages and may increase the stiffness of the mixture. In terms of heating efficiency, metallic fillers appear to offer greater advantages compared to carbon-based fillers.

From a cost-effectiveness perspective, industrial by-products such as steel slag can provide moderate improvements in electrical conductivity at a relatively low cost. Therefore, the optimal selection of conductive fillers should consider the conductivity enhancement per unit content, the retention of mechanical strength, and economic feasibility. A comparison of commonly used conductive fillers in ECAC systems is shown in Table 4.

**Table 4:** Comparative evaluation of conductive fillers in ECAC.

Filler Type	Typical Dosage	Percolation Threshold	Conductivity Improvement	Mechanical Impact	Cost Level	Reference
Graphite	5–15 wt.%	>10 wt.%	High	Slight stiffness increase	Low	[118]
Carbon nanotubes	0.5–2 wt.%	0.5–1 wt.%	Very High	High	Very High	[119]
Carbon fiber	0.3–1.0 vol.%	5.5 wt.%	Very high	Improves fatigue	High	[120]
Steel fiber	1–3 vol.%	<18 vol.%	High	an increase of 27% in ITS value	Medium	[112]
Steel slag	-	15 wt.%	Moderate	Improves rutting	Low	[121]
Graphene nanoplatelets	<10 wt.%	7 wt.%	High	Minor improvement	High	[122]

### 4.2. Design and Optimization of Mix Proportion for ECAC Mixture

#### 4.2.1. Relationship Between Conductive Filler Content and Conductivity

**Figure 11:**
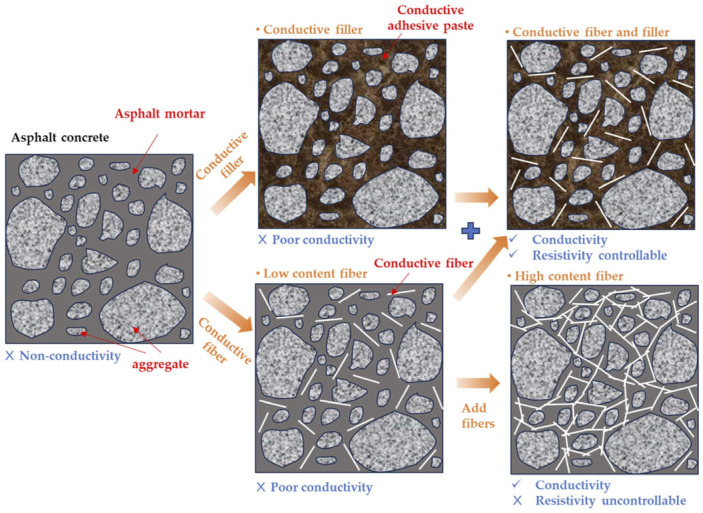
Schematic Diagram of the Meso-Microstructure and Conductive Performance Regulation Mechanism of Conductive Asphalt Concrete in Different Modification Systems [68].

Conductive filler dosage critically governs asphalt’s transition from insulating to conductive states. Percolation theory explains that once the filler volume fraction reaches the percolation threshold, resistivity drops by several orders of magnitude as isolated particles or fibers form a continuous, long-range conductive network [123]. In de-icing applications, precise control of asphalt resistivity is critical for energy efficiency and operational safety [124,125]. Exceeding the percolation threshold alone is insufficient; hybrid filler selection and dosage must be optimized to enable gradual, controllable resistivity transitions, ensuring stable heating and minimized energy consumption [126]. Figure 11 illustrates the process of regulating the electrical conductivity of asphalt mixtures by adding conductive fibers: On the left, conventional asphalt exhibits poor overall conductivity and uncontrollable resistivity due to non-conductive aggregates and low fiber content; As fiber content increases, intermediate states begin to form dispersed conductive pathways; On the right, when fiber content reaches a critical threshold, fibers interconnect to form a complete conductive network, achieving stable conductivity and controllable resistivity [68].

Extensive studies have demonstrated that conductive fibers with higher aspect ratios generally exhibit superior performance compared to granular fillers, as their elongated geometry significantly increases the probability of establishing continuous conductive pathways [127]. Furthermore, the synergistic integration of fibers and particulate fillers can markedly enhance the connectivity and robustness of the conductive network. In this hybrid system, the particles facilitate the formation of localized, short-range clustering structures, while the fibers function as structural “bridges” that interconnect these clusters. This collaborative interaction effectively constructs a multi-scale conductive network, optimizing electron transport across both micro- and macro-dimensions [128].

However, exceeding the optimal dosage range of conductive fillers often leads to “conductive path saturation”, which diminishes sensing sensitivity, elevates material costs, and compromises both volumetric stability and mechanical performance. For instance, incorporating high dosages exceeding approximately 15 wt.% graphite or 20 wt.% carbon black (by weight of the asphalt binder) has been shown in certain studies to significantly impair the thermal cracking resistance of asphalt mixtures. Research indicates that the optimal dosages for advanced nano-fillers are considerably lower: graphene typically requires approximately 0.65 wt.%, whereas carbon nanotubes (CNTs) achieve peak functional efficiency at around 1 wt.% [97,129]. Maintaining these precise thresholds is critical to preventing the agglomeration of nanomaterials, which can otherwise act as stress concentration sites and trigger premature structural failure.

#### 4.2.2. Synergistic Effects of Grading and Oil/Stone Ratio on Mechanical and Electrical Conductivity Properties

Aggregate gradation plays a pivotal role in asphalt mixture design, significantly influencing volumetric characteristics, mechanical properties, and long-term durability [130]. For instance, gradations engineered via the Bailey method exhibit exceptional stability and rutting resistance, attributed to optimized aggregate packing and enhanced particle interlocking [131]. Within these mixtures, coarse aggregates typically constitute over 60 wt.% of the total composition, establishing a robust skeletal framework that serves as the primary mechanism for transmitting and dissipating traffic-induced stresses [132].

**Figure 12:**
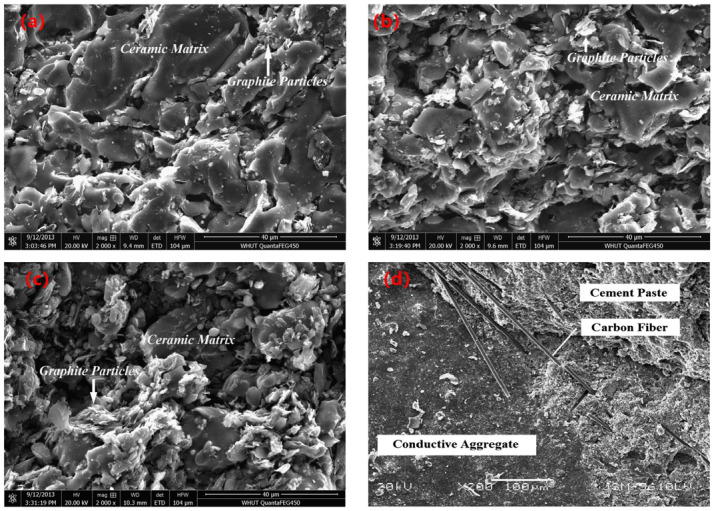
SEM Morphology of Conductive Aggregates with Different Graphite Contents and Their Interfacial Zones: (**a**) SEM image of conductive aggregate containing 6% graphite; (**b**) SEM image of conductive aggregate containing 10% graphite; (**c**) SEM image of conductive aggregate containing 20% graphite; (**d**) SEM image of the interface region between conductive aggregate and cement paste [133].

Although a unified gradation standard for ECAC is lacking, maintaining sufficient voids in mineral aggregate (VMA) is crucial to accommodate conductive fillers while preserving mechanical integrity. Replacing conventional aggregates with conductive powders or industrial by-products, such as steel slag, can alter volumetric density, necessitating careful evaluation to prevent structural degradation [82]. As shown in Figure 12a–c, increasing the graphite content from 6% to 20% gradually promotes the formation of a conductive network. At 6%, graphite particles are sparsely dispersed within the ceramic matrix without effective interconnection. At 10%, partial particle contacts and chain-like structures appear, indicating the approach to the percolation threshold. At 20%, a continuous graphite network is clearly formed, providing stable electron transport pathways. Figure 12d shows a relatively dense interfacial transition zone between the conductive aggregate and cement paste. Carbon fibers bridge across the interface, contributing to both electrical connectivity and structural integrity.

The aggregate skeleton provides both mechanical stability and a template for conductive network formation [134]. Conductive aggregates, such as steel slag, bridge isolated pathways and shorten electron transport distances. Aggregate packing governs filler distribution and network connectivity, while asphalt binder content balances conductivity and mechanical cohesion [133,135]. Mix designs should therefore optimize gradation and binder thickness to ensure both structural integrity and electrical performance.

### 4.3. Mechanical Properties and Durability of ECAC

#### 4.3.1. High-Temperature Stability, Low-Temperature Crack Resistance, and Water Stability

ECAC mixtures exhibit enhanced compactability, reduced air voids, and up to 40–60% higher dynamic stiffness as reported in [136], indicating improved load distribution. Conductive fillers—graphene, graphite, magnetite—enhance fatigue life, rutting resistance, and high-temperature stability [137,138,139]. Even low graphene contents (~0.65 wt.%) significantly improve binder deformation resistance, while fine powders increase asphalt softening points by ~40 °C. Steel fibers boost Marshall stability and shear resistance, and steel slag aggregates improve high-temperature skeletal stability [139]. SBS modifiers further optimize performance.

Conductive fillers—graphene, graphite, magnetite, steel fibers, steel slag, and carbon black—enhance asphalt high-temperature stability and rutting resistance. This is achieved through increased dynamic stiffness, higher compaction density, elevated binder softening points, and the formation of an interconnected filler skeleton that reinforces the mastic, resisting shear deformation under heavy traffic at elevated temperatures [121,136,137,138,139].

**Figure 13:**
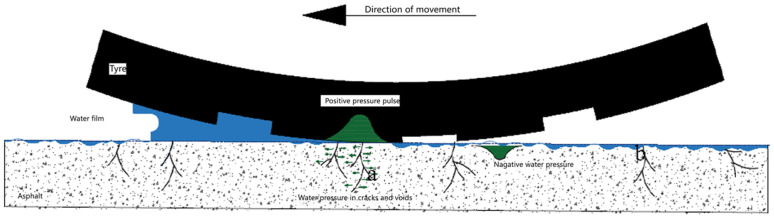
Conceptual diagram of tire–water–road interaction [141].

Low-temperature cracking is a predominant pavement distress in cold climates, severely compromising both functional performance and structural lifespan [140]. The influence of conductive fillers on low-temperature properties is multifaceted and composition-dependent. Research indicates that graphene maintains a negligible impact on thermal cracking resistance when incorporated at optimized dosages (up to 0.65 wt.%) [97], whereas steel fibers significantly bolster low-temperature fracture toughness [29]. Concurrently, moisture-induced damage remains a critical durability concern for asphalt pavements. This phenomenon, typically triggered by moisture infiltration, facilitates the stripping of the binder from the aggregate surface, leading to strength degradation, spalling, delamination, and progressive aggregate loosening [137]. Under the load of vehicles, moisture is forced into the interior of the asphalt mixture, causing irreversible damage, as shown in Figure 13.

The effects of conductive fillers on asphalt moisture stability are material-dependent. Graphite often reduces water resistance, while steel slag (SS) and copper slag (CS) may increase expansion, porosity, or water absorption, compromising durability [82,142]. Conversely, combining steel slag with blast furnace slag (BFS) improves moisture resistance [143]. Anti-stripping agents, such as hydrated lime or cement, are commonly used to reinforce interfacial bonding and enhance overall water stability [144].

#### 4.3.2. Fatigue Performance and Wear Performance

Fatigue cracking in asphalt arises from cumulative damage under repeated traffic loading [145]. ECAC mixtures can retard crack propagation through conductive fillers such as carbon-based materials and metallic fibers. Steel fibers enhance fatigue resistance via a bridging effect, while graphene shows moderate improvement at optimized dosages (0.65 wt.%) [97]. When steel slag is used as a conductive aggregate, fatigue life varies non-linearly with content; excessive slag increases stiffness and internal stress, potentially reducing durability [146].

Conductive fillers typically enhance the stiffness of mixtures and improve fatigue life to some extent [147]. However, excessive stiffness reduces material flexibility, making it more susceptible to fatigue cracking at moderate temperatures [148]. For instance, the addition of 0.5% carbon fiber increases the fatigue life of the mixture by approximately 51% at 40 °C, enhances the stiffness modulus (ITSM) by a factor of 1.38–1.51 [149]. At a reference temperature of 25 °C, it exhibits nearly 200% lower tensile strain levels compared to conventional road surfaces (5–7 με vs. ~14 με), thereby improving the fatigue performance [150]. 18% graphite conductive asphalt concrete exhibits a higher fatigue life than conventional concrete when the stress exceeds 0.6 MPa; the addition of 2% carbon fiber further increases the fatigue life [151].

The wear resistance of asphalt pavements directly determines their service life and quality, influenced by factors such as compressive strength, aggregate properties, and maintenance standards [152]. Studies indicate that higher compressive strength generally enhances wear resistance, while additives like fiber materials and silica powder can also improve surface durability [153].

#### 4.3.3. Stability and Long-Term Service Performance of Conductive Networks

**Figure 14:**
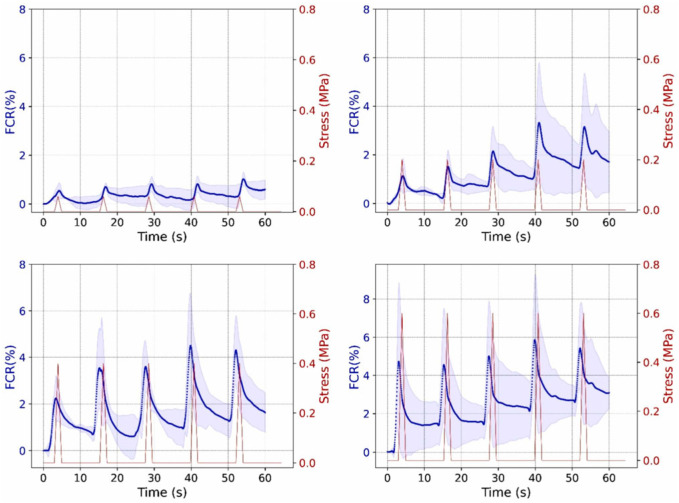
Time histories of FCR (blue solid line) and stress (red dashed line) under cyclic loading conditions, showing the progressive damage behavior of the asphalt mixture specimen [156].

The self-sensing and de-icing performance of ECAC depends on the stability and responsiveness of its internal conductive network. Electrical properties are determined by the geometry, composition, and dosage of conductive additives. External factors—mechanical stress, temperature, humidity, and material damage—can disrupt network continuity, causing resistivity changes that underpin sensing functions [77]. ECAC often shows gradual attenuation of fractional resistance change (FCR) and a declining gauge factor (GF) under cyclic loading, due to irreversible plastic deformation and microstructural degradation [154]. Low-graphite composites, however, maintain stable GF over multiple cycles, indicating that long-term piezoresistive reliability depends on both conductive network topology and material composition [155]. Figure 14 demonstrates that under cyclic loading, the electrical conductivity (FCR) of conductive asphalt concrete exhibits synchronous periodic responses with stress variations, proving that this material can serve as a self-sensing pavement for real-time monitoring of traffic loads [156].

ECAC exhibits thermistor behavior, with resistivity varying in response to temperature due to changes in carrier density and mobility [155]. Most composites show a positive temperature coefficient (PTC), where resistivity rises with thermal expansion that increases inter-particle spacing. Certain systems, especially those with cementitious or carbon-based phases, display a negative temperature coefficient (NTC), as thermal fluctuations enhance tunneling and carrier hopping [157].

Conflicting reports on PTC and NTC effects in ECAC stem primarily from variations in filler type, concentration, dispersion quality, and testing temperature ranges [157]. In most carbon-based ECAC systems, PTC behavior predominates because thermal expansion of the asphalt matrix increases inter-particle gaps, suppressing quantum tunneling and field emission [158]. In contrast, NTC is typically observed in highly dispersed nano-filler or cementitious hybrid systems where temperature rise enhances carrier mobility and hopping [159]. This variability can impair energy management and current control in active de-icing systems, potentially causing suboptimal heating (under PTC) or localized overheating (under NTC), thereby compromising reliability and safety. To mitigate these inconsistencies, hybrid filler strategies (e.g., carbon fiber + graphite) and intelligent temperature-compensating control systems are recommended, alongside standardized testing protocols across temperature ranges relevant to winter service conditions [160].

Long-term aging under repeated heating cycles—critical for practical de-icing applications—has received limited attention in early studies but reveals progressive degradation. Thermal-oxidative aging from repeated Joule heating accelerates binder hardening and volatilization, leading to gradual resistivity drift and reduced heating efficiency (after 30 freeze–thaw cycles, thermal conductivity and specific heat decreased by 12.7% and 20.8%, respectively) [161,162]. This also reduces heating efficiency and exacerbates mechanical deterioration such as fatigue cracking and loss of low-temperature toughness [163]. Filler migration and interfacial debonding further destabilize the conductive network under sustained electrothermal stress. Long-term stability of the conductive network is further challenged by oxidation, freeze–thaw cycles, and traffic-induced microcracking. Freeze–thaw cycles induce micro-cracking at filler–matrix interfaces and aggregate debonding, causing conductivity loss of 20–50% after 50–100 cycles due to disrupted conductive pathways [164]. Traffic-induced microcracks from repeated loading propagate along weak interfaces, further fragmenting the network and exacerbating FCR/GF attenuation over time. These combined effects can compromise both self-sensing accuracy and de-icing reliability. To enhance long-term durability, incorporation of anti-oxidant additives, thermally stable filler coatings, and laboratory protocols simulating field heating cycles are strongly recommended [165].

Moisture infiltration weakens binder–aggregate adhesion, accelerating structural distress, while its effect on electrical properties remains largely unexplored [166]. Water can disrupt conductive networks, and repeated thermal cycles induce micro-cracking and interfacial debonding, abruptly increasing resistivity. The asphalt’s viscoelasticity further amplifies sensitivity to temperature fluctuations, causing significant conductivity instability [167].

## 5. Design and Application of ECAC Deicing and Snowmelt System

### 5.1. Composition of ECAC Deicing and Snowmelt System

#### 5.1.1. ECAC Pavement

**Figure 15:**
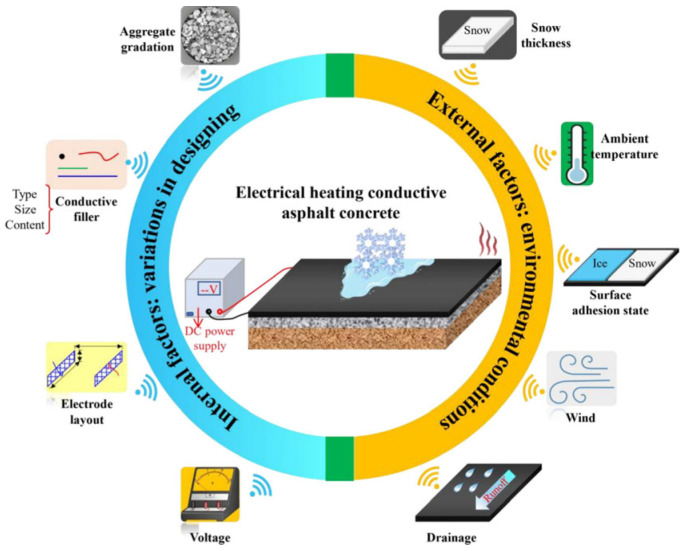
Schematic diagram of influencing factors on snow-melting performance of electrically heated ECAC [168].

ECAC is engineered by integrating conductive additives into the asphalt matrix [138]. While pristine asphalt is a dielectric insulator [36], strategic filler incorporation drastically reduces resistivity, enabling Joule heating-induced snow-melting and de-icing. Key factors influencing the snow and ice melting performance of electrically heated conductive asphalt concrete include aggregate gradation, conductive filler, and electrode layout at the material design level, as well as snow depth, ambient temperature, wind speed, drainage conditions, and surface adhesion status at the environmental operating level. Voltage, as the input energy parameter, plays a regulatory role. This demonstrates that electrically heated pavement is a complex system influenced by multiple coupled factors, as shown in Figure 15 [168].

Furthermore, the conductivity and mechanical properties of ECAC are highly dependent on the type, dosage, and dispersion state of the selected conductive additives. Different conductive materials exhibit varying network-forming capabilities and reinforcement effects, and their impact on overall performance must be comprehensively considered.

#### 5.1.2. Power Supply System and Control Unit

**Figure 16:**
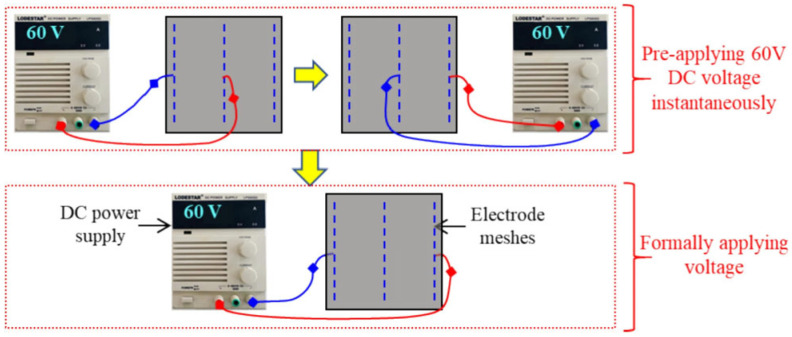
Schematic diagram of pre-voltage application method and formal voltage application [168].

In heating-type transportation infrastructure systems, 24 V alternating current (VAC) is recommended as the operating voltage to comply with the safety standard of <30 VAC for electrical equipment in public places under humid conditions, thereby minimizing electric shock risk [60]. In contrast, laboratory and controlled-field studies often employ 60 V to achieve efficient heating performance (higher power output via P = U^2^/R), as illustrated in Figure 16 [168]. This apparent contradiction reflects a fundamental trade-off between safety and heating efficiency: while 24 VAC ensures compliance in open public areas, 60 V enables faster de-icing in insulated or electrode-protected systems. In practice, the conflict is resolved through isolation transformers or step-up/step-down converters that deliver safe low voltage at the supply end while optimizing power delivery to the conductive layer [73,163].

System energy consumption is heavily influenced by structural and geometric configurations. Studies indicate that increasing electrode spacing can significantly reduce energy demand—by up to 50%—while maintaining temperatures above freezing [169]. Conversely, conductive layer thickness inversely affects efficiency; thinner layers may consume substantially more energy to achieve comparable de-icing performance [170]. These findings highlight the need to optimize the aspect ratio of the conductive volume and electrode placement to balance heating responsiveness with energy conservation.

Optimizing conductivity, electrode configurations, and operating voltage is essential to minimize energy footprint. Notably, each system exhibits a “threshold voltage”; exceeding this limit can paradoxically impair heating efficiency and increase energy consumption [171]. Thermodynamically, voltages above the threshold cause excessively rapid surface temperature rise, leading to disproportionately higher heat losses to the ambient environment through convection and radiation, thereby reducing the fraction of input electrical energy that is effectively converted into latent heat for ice melting [88,172]. Modern systems leverage advanced control units for dynamic energy allocation. Specifically, Programmable Logic Controller (PLC) technology enables remote, real-time diagnostics with robust field adaptability [173,174]. Furthermore, “proactive de-icing”—activation prior to snowfall—is the optimal operational paradigm. By preventing the ice-pavement bond, this strategy significantly curtails snow accumulation and total energy expenditure [175].

Smart grids enhance ECAC operational efficiency by facilitating renewable energy integration and dynamic load management. By providing real-time data on generation and consumption, these grids enable optimized scheduling for high-power-density scenarios like heated pavements [173]. Through demand response (DR) mechanisms, operators can dynamically adjust usage based on price signals or utility incentives. This strategy mitigates peak demand and shifts consumption to off-peak periods [176], thereby alleviating grid instability and reducing the capital costs associated with generation capacity expansion [177].

Furthermore, energy storage systems—particularly vehicle-to-grid (V2G) technology—can store surplus renewable energy during off-peak hours and release it during peak demand, thereby enhancing grid load balancing, operational stability, and energy efficiency [176].

#### 5.1.3. Sensors and Intelligent Monitoring System

The intelligence of ECAC systems relies on an integrated sensor network for autonomous data acquisition. Meteorological instruments monitor humidity, temperature, and wind speed to provide inputs for icing prediction models and thermal optimization [178,179,180]. Concurrently, embedded sensors capture internal stress, strain, and deformation data, offering diagnostic insights for Pavement Management Systems (PMS) and structural integrity assessment [181]. Precise thermal monitoring via thermocouples or fiber optic sensors enables real-time feedback control, preventing localized overheating and thermal degradation during heating cycles [182].

**Figure 17:**
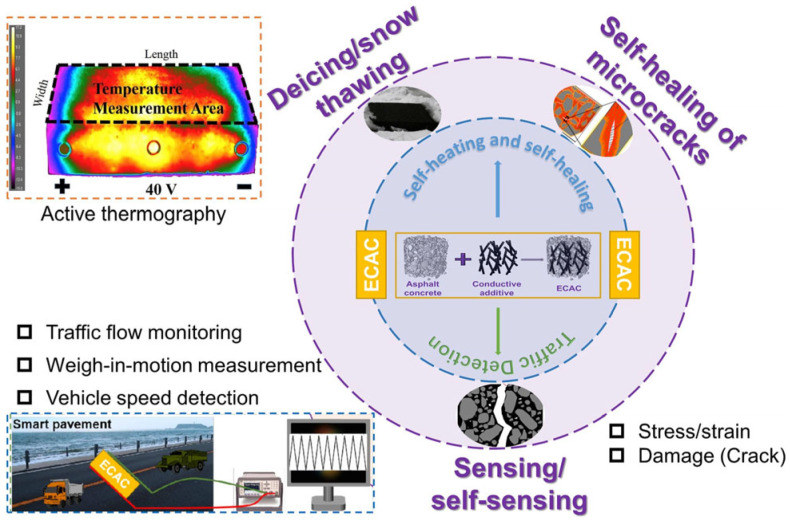
Multifunctional electrothermal conductive asphalt concrete (ECAC) for intelligent and durable pavement systems: schematic illustration of active thermography for deicing/snow-thawing, self-healing of microcracks, self-sensing for traffic monitoring and structural health monitoring, and their integrated applications [77].

The ECAC exhibits intrinsic piezoresistive properties, enabling self-sensing of structural deformation and micro-damage without external instrumentation [117]. To complement this, integrated piezoelectric sensors can quantify icing thickness via specialized response analysis; these sensors demonstrate high durability under repetitive vehicular loading and extreme temperatures [178]. Furthermore, wireless MEMS and NEMS technologies provide cost-effective Structural Health Monitoring (SHM). Notably, self-powered wireless architectures facilitate continuous data acquisition and autonomous storage, ensuring long-term diagnostic reliability for pavement systems [183]. Figure 17 demonstrates the multifunctional applications of electrically conductive asphalt concrete (ECAC): enabling self-healing of micro-cracks and snow/ice melting, along with intelligent pavement applications such as traffic flow monitoring, dynamic weighing, and vehicle speed detection [77].

Despite their potential, sensor integration faces durability and cost barriers [184]. A primary technical challenge is the stiffness mismatch between rigid encapsulation modules and the viscoelastic asphalt matrix, which can compromise both sensing accuracy and pavement integrity. Developing sensors with matched stiffness and high sensitivity remains a priority [185]. Meanwhile, self-aware pavements—capable of modulating electrical properties—offer transformative potential for Structural Health Monitoring (SHM), Digital Twin modeling, and V2I communication. Techniques like impedance spectroscopy and infrared thermography are essential for evaluating stability and thermal efficiency under cyclic loading [186]. Leveraging self-powered sensing and data-driven algorithms allows authorities to move toward proactive maintenance, identifying early damage and forecasting residual fatigue life [187,188].

### 5.2. Key Technologies in Engineering Applications

#### 5.2.1. Construction Techniques and Quality Control

The field performance of ECAC is heavily contingent upon material proportions and mixing protocols [189]. Ensuring uniform dispersion is paramount, as excessive conductive fibers (e.g., carbon fibers) trigger agglomeration, paradoxically compromising both connectivity and mechanical stability [190]. Consequently, stabilizing electrical resistivity during construction and service remains a primary challenge [191]. ECAC design requires a delicate equilibrium between functional conductivity and structural durability, as additives often involve trade-offs in rutting resistance, fracture toughness, and moisture susceptibility [192]. Although localized electrode embedding is a perceived vulnerability, empirical data show structural integrity comparable to conventional mixtures [14]. However, the lack of standardized technical guidelines remains the principal barrier to large-scale commercial production.

To ensure construction quality and serviceability, non-destructive testing (NDT) and advanced quality control are imperative. Three-dimensional Ground-Penetrating Radar (GPR) and electromagnetic density gauges enable the characterization of air void distribution and internal anomalies by analyzing dielectric variance [193,194]. Such rapid assessment of compaction uniformity is critical, as excessive porosity triggers moisture damage and premature aging [195]. Additionally, Intelligent Compaction (IC) technology—integrating GPS and real-time sensors—allows for the immediate detection and correction of under-compacted zones during laydown [196]. Finally, field verification of electrical resistance is essential to confirm that the conductive network satisfies design specifications for de-icing or self-sensing functionality [60].

Long-term maintenance is vital to ensure both de-icing functionality and structural integrity. Cracks propagating perpendicular to electrodes are of particular concern, as they create high-impedance zones and leakage risks, necessitating rapid remediation for operational safety [60]. While dielectric coatings provide a safety barrier, they require periodic reapplication due to traffic abrasion [197]. To optimize interventions, Pavement Management Systems (PMS) leverage real-time data to simulate degradation and trigger proactive maintenance, which is critical for extending service life [198]. Furthermore, combining embedded sensors with infrared thermography enables high-fidelity monitoring of slabs, facilitating the performance assessment of diverse designs during actual snow and ice events [199].

#### 5.2.2. Electrical Connection and Safety Protection

**Figure 18:**
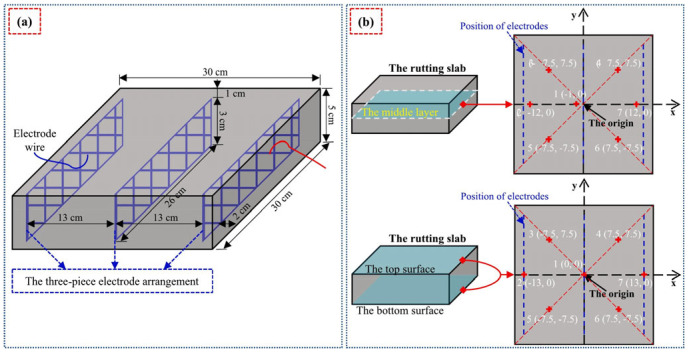
Schematic diagram of electrode installation: (**a**) Three-piece electrode arrangement, (**b**) Three-layer thermocouple arrangement [168].

Electrodes are central to ECAC systems, converting electrical current into Joule heat. While steel bars are common [170], perforated steel plates are recommended for their superior interfacial bonding and electrical connectivity [200]. Electrode spacing significantly impacts thermal efficiency; for example, reducing spacing from 30 cm to 15 cm can increase surface temperatures by 1.7 °C while halving energy consumption [169]. Additionally, burial depth is critical: a 7.5 cm depth attenuates heating efficiency, whereas shallower placement mitigates low-temperature thermal cracking [170,201]. Although larger electrodes augment energy conversion, flat or small-diameter variants are often preferred for cost-effectiveness [174]. For long-term reliability, designs must specify overcurrent protection, grounding, and waterproof junction boxes (IP-rated) with robust drainage to prevent moisture-induced short circuits and corrosion [202]. Figure 18 illustrates the electrode arrangement in conductive asphalt concrete: Figure 18a shows a three-electrode configuration, with electrode wires embedded at 5 cm intervals within a 30 cm wide specimen to form a conductive circuit by applying voltage; Figure 18b shows a three-layer thermocouple arrangement.

Electrical safety is a foundational constraint for ECAC systems, which operate in humid, salt-rich environments prone to electric shock and thermal risks. Since human body impedance fluctuates with humidity and contact area, simultaneous contact with energized panels can trigger shock incidents [203]. According to NEC and UL standards, the safety threshold is 30 VAC for dry conditions and 15 VAC for wet environments. Although specific regulations for ECAC are not yet finalized, these infrastructures are currently categorized as “electrical products for wet locations” during compliance assessments [60].

Grounding is vital for operational integrity, requiring all metallic components—frames, enclosures, and conduits—to be bonded via low-impedance pathways. Grounding electrode conductors, typically #3/0 bare stranded copper, must be routed through rigid PVC conduits to avoid induced current interference [204]. These systems should be integrated with the foundation reinforcement prior to concrete pouring. Notably, some research suggests an “ungrounded” (IT) power system to enhance safety by minimizing initial fault currents during single-phase insulation failures [205].

Safety devices such as ground fault circuit interrupters (GFCIs) are typically configured to trip automatically when 5 mA current passes through a person [206]. While applying protective coatings to ECON panels can serve as an auxiliary electrical safety measure, it should not be considered the primary safety solution due to the need for regular maintenance to prevent concrete exposure.

### 5.3. Case Analysis of ECAC Deicing and Snowmelt Engineering

**Figure 19:**
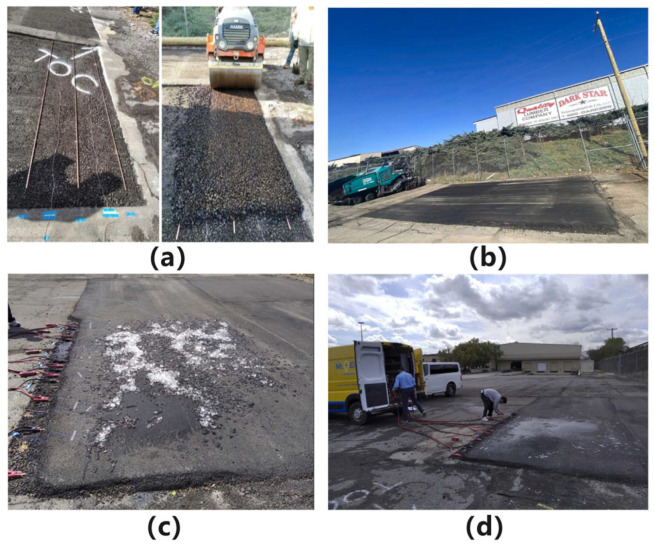
Construction and Heating Performance Testing of In Situ Electrically Heated Pavement: (**a**) Construction of conductive surface layer on three electrodes; (**b**) Application of conductive surface layer; (**c**) At the start of de-icing test; (**d**) After de-icing test completion [207].

Wang et al. [207] reported a full-scale field implementation of coal-based ECAC pavement, consisting of three construction stages (Figure 19). After milling the existing surface, a 3.8 cm conventional HMA leveling layer was placed and compacted. The conductive functional layer was then constructed by installing copper rod electrodes (1.27 cm in diameter, 3.25 m in length) at predefined spacing, followed by placement and compaction of a coke-based conductive cold-mix asphalt, yielding a conductive layer approximately 3.0 cm thick. Electrode–mixture resistance was continuously monitored to confirm the formation of a stable conductive network. Finally, a 3.8 cm HMA 411-D mixture was applied as a sacrificial wearing course and dielectric insulation layer. For comparison, non-conductive control sections were paved with a single 6.8 cm thick layer of the same HMA mixture. In laboratory tests, the system achieved a surface temperature rise from −5 °C to 8.3–11.7 °C with a power density of 473 W/m^2^; field tests demonstrated complete snow/ice melting within 2–4 h depending on snow depth and power input (Figure 19).

**Figure 20:**
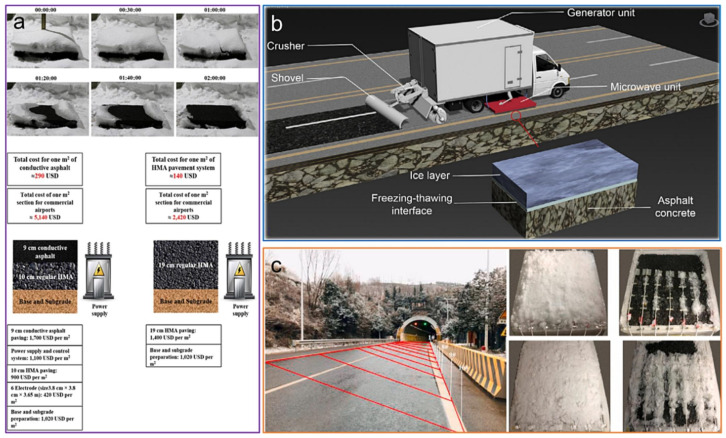
ECAC and its application in asphalt pavement anti-icing and de-icing: (**a**) De-icing capacity and cost estimation (slab size: 4.5 m × 3.8 m); (**b**) Microwave de-icing using steel slag in ECAC; (**c**) Changes in snow layer at the exit of Jiangjunling Tunnel and during heating at −3 °C [90].

Despite its functional advantages, ECAC entails a substantial economic trade-off, with construction costs approximately 212% higher than those of conventional pavements [208]. To address this limitation, Gao et al. [209] demonstrated that replacing 40–60 vol.% of conventional aggregates with steel slag in microwave-assisted ECAC significantly improves winter road safety. Their life-cycle assessment, incorporating material costs, logistics, and environmental impacts, confirmed steel slag as a cost-effective and high-performance alternative (Figure 20b). Similarly, Jiao et al. [210] reported that the superior thermal properties of steel slag increased snow-melting efficiency by 25–38% while maintaining mechanical performance within specification limits. Future research should therefore focus on increasing the volumetric content of functional aggregates and optimizing their spatial distribution to enhance internal heat generation and interlayer thermal conductivity, thereby improving the responsiveness of anti-icing and de-icing systems in field applications.

**Figure 21:**
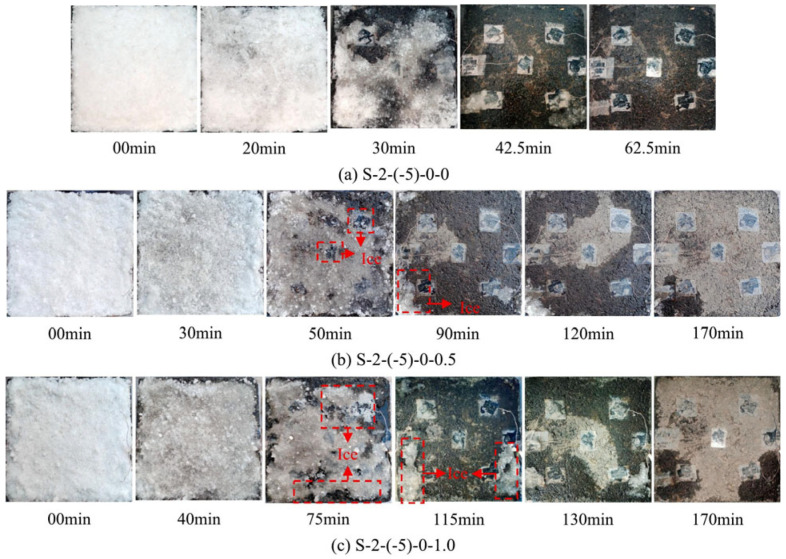
Snow-melting behavior of modified ECAC under varying wind speeds [168].

Zhang et al. [168] investigated the snow-melting kinetics of ECAC under different convective wind conditions using high-resolution photographic monitoring (Figure 21). Under low wind conditions, specimen S–2–(−5)–0–0.5 achieved a 92.65% reduction in snow mass within 90 min and approached complete melting after 170 min. By contrast, at higher wind speeds, specimen S–2–(−5)–0–1.0 required 115 min to reach a comparable mass reduction (92.35%), although near-complete melting was attained at the same heating duration. These results quantitatively demonstrate the inhibitory effect of wind-induced convective heat loss on snow-melting efficiency, which was captured by adjusting the observation time windows to 90 and 115 min for the two test conditions.

To provide a comprehensive overview of key performance metrics across representative ECAC case studies, the major indicators—including energy consumption, snow-melting time, service life, and construction/maintenance costs—are summarized in Table 5.

## 6. Conclusions

Electrically conductive asphalt concrete (ECAC) enables active snow melting and de-icing through the Joule heating effect, enhancing the safety of transportation infrastructure in cold regions while effectively avoiding the environmental and structural corrosion issues caused by chloride-based deicers. Significant progress has been made in existing research regarding the selection of conductive fillers, mix design optimization, and validation of snow-melting performance, demonstrating the engineering applicability of this technology in heavy snow and ice regions. However, for ECAC technology to transition from laboratory research to large-scale engineering applications, the following key scientific challenges and technical bottlenecks must be addressed:

(1) Under actual service conditions involving vehicle dynamic loading, temperature cycles, and water erosion, the contact state and spatial topology of conductive fillers within ECAC undergo irreversible evolution, leading to resistivity drift and attenuation of Joule heating efficiency. Current research has yet to establish quantitative models capable of describing such time-dependent degradation behavior of the conductive network, which directly constrains accurate prediction of system service life. Future efforts should focus on developing conductive network evolution models based on percolation theory and microstructural characterization, to reveal the failure mechanisms of conductive pathways under coupled load–temperature–moisture conditions.
(2) Although the incorporation of conductive fillers enhances electrothermal efficiency, it often adversely affects the low-temperature crack resistance or moisture stability of the mixture. Existing studies predominantly focus on single-performance evaluations, lacking systematic understanding of the trade-off mechanisms between electrical conductivity and pavement performance. Future research should establish multi-scale constitutive relationships that comprehensively consider conductive filler distribution, temperature field evolution, and mechanical response, providing theoretical foundations for material design and structural optimization.
(3) Most existing studies employ relatively high voltages to ensure heating rates, yet insufficient attention has been paid to electrical safety concerns in wet pavement environments. Achieving rapid and uniform snow melting and de-icing under human-safe voltage conditions remains a core challenge that must be resolved for practical ECAC applications. Future research should prioritize the development of novel composite conductive material systems with high electrothermal conversion efficiency at low voltages, combined with energy management and real-time monitoring technologies, to integrate on-demand heat supply with system safety protection.

## Figures and Tables

**Table 1:** Comparative Analysis of Deicing Technologies (Traditional Method vs. ECAC).

Feature	Conventional Method	ECAC
Method type	Passive, externally administered	Active integrated infrastructure
Main mechanism	Lowering the freezing point, physical removal	Road electric heating
Environmental effects	High	Low
Infrastructure impact	High	Low/Positive
Service life	Shorten	Expected to last longer
Operational efficiency	Labor-intensive and resource-intensive, with high hidden costs, and limited applicability	Automation, continuous operation, clear objectives, consistent products
Security upgrade	Increased traction, but reduced visibility, driver inexperience, and damaged infrastructure pose risks	Improving safety through continuous road cleaning, particularly for critical infrastructure
Application area	General roads, budget/resource constraints	Key areas (airports, bridges), high-traffic roads, smart city infrastructure
Maintenance costs (Based on a 20-year lifecycle)	1 (Benchmark)	2%
Energy consumption (Based on a 20-year lifecycle)	1 (Benchmark)	71%
Annual Expenditure Funds	1 (Benchmark)	73%
Cost Overview	Salt has a low initial unit cost, but high hidden/lifecycle costs	High initial investment, but potential long-term savings

**Table 2:** Common non-chlorinated salt de-icing agents.

Type	Usability	Consult
Acetate	The most commonly used are potassium acetate (KAc), calcium magnesium acetate (CMA), and sodium acetate (NaAc). They are widely commercialized.	[43]
Formate	The most common compounds are sodium formate (CHO_2_Na) and potassium formate (CHO_2_K). These substances are in limited supply and expensive, primarily used for de-icing at airports.	[44]
Urea CO(NH_2_)_2_	It was widely used in airport de-icing, but gradually replaced in winter highway maintenance due to its great impact on the environment.	[45]
Glycerol/ethylene glycol products	Primarily used in airports, but less frequently in winter highway maintenance	[46]
Succinate	The most commonly used is potassium succinate (C_4_H_4_O_4_K_2_), a liquid de-icing agent comparable to potassium acetate.	[47]
Additive	They are typically used in small quantities in combination with de-icing agents, such as organic products like beet juice, molasses, wine lees soluble solids, and corn syrup. These substances do not melt snow themselves but can serve as additives for pre-wetting agents or antifreeze agents.	[43]
Abrasive (sand)	It does not melt snow or ice, but provides short-term traction on snow or ice. In extremely cold temperatures, when de-icing agents are ineffective, abrasives are the only effective product.	[43]

**Table 3:** Performance, Cost and Impact of Common Deicing Agents [53].

Class	Material Name	Effective Snow-Melting Temperature Range	Corrosiveness of Steel	Influence on Concrete	Environment Effects	Prime Cost	Main Application Scenarios
Chlorides	Sodium chloride (rock salt/saltwater)	Above −17.8 °C (saltwater); as low as−23.14 °C (treated salt)	High	Erosion, salt crystallization, and aggravated freeze–thaw damage	Long-term accumulation, water/soil pollution, plant damage, BOD	Low dollar rate: 30 to 80/ton	extensive highway maintenance
	Calcium chloride	Most effective at lower temperatures, exothermic	High	Formation of chloro compounds, severe cracking	Accumulation, water/soil pollution	Medium dollar rate: 150 to 300/ton	Airport area, such as bridges
	Magnesium chloride	One of the best de-icing agents, with a low freezing point	High	Severe cracking (hydromagnesite, M-S-H, etc.)	Accumulation, water/soil pollution	Medium dollar rate: 120 to 250/ton	Specific areas, such as bridges
Non-chloride salts	Potassium Acetate (KAc)	−35 °C to 0 °C	High	On the influence of Concrete, inducing ASR	High BOD, no accumulation	High dollar rate: 1000 to 1500/ton	Airports, bridge decks, Salt-free areas
	Sodium formate	−15 °C to 0 °C	Low	Not specified	Minimum BOD, no accumulation	High dollar rate: 1800 to 2200/ton	Airdrome
	Urea	−9.4 °C to 0 °C	Low	Not specified	Dissolves dissolved oxygen without accumulation	High dollar rate: 1500/ton	Airdrome
	Glycerol ethylene glycol	−15 °C to 0 °C	Low	Not specified	High BOD, difficult to recover, toxic ethylene glycol	High dollar rate: 2297 to 4594/ton	Airdrome
	Sodium succinate	−5 °C to 0 °C	Non-corrosive, mitigates saltwater corrosion	Concrete spalling occurs very rarely	The BOD value is similar to that of acetate.	High (with acetate)	Airport (Emerging)
	Sand (Abrasives)	Any temperature (no snowmelt)	Not have	Not specified	Dust generation and drainage obstruction	Low	Provide traction

**Table 5:** Performance Comparison of Representative ECAC Case Studies.

Parameters	Numerical Value	Conditions	References
Energy consumption	215 W/m^2^	Design target power for effective snow melting (1960s experiments)	[211]
	358–430 W/m^2^	Laboratory ice melting, maintaining surface temperature above 0 °C	[212]
	1623 W/m^2^	Laboratory, rapid temperature rise from −20 °C to 0 °C (128 s)	[208]
	500–750 W/m^2^	Reference Values for Traditional Electric Snow Melting Systems (Based on Application Experience in West Virginia/Oregon)	[213]
Annual energy consumption	24.6–1444.8 kWh/m^2^	Annual simulation (Pittsburgh mild climate: 25 kWh/m^2^; Fairbanks severe cold: 1445 kWh/m^2^), under smart control	[212]
Snowmelt Time	120–150 min	Laboratory crushed ice, ~400 W/m^2^, ambient temperature −10 °C	[212]
	45 min	On-site, 8 cm of snow accumulation, ambient temperature −1 °C	[208]
	120 min	Steel fiber conductive asphalt, 269.5 W/m^2^, reference for cold regions	[214]
Service life	Observations over two winters (resistivity increased by 21–83%)	No traffic load, graphite-modified conductive asphalt	[211]
	Mechanical properties meet specifications, with an expected service life of 10–20 years or more.	Carbon fiber/steel slag admixture improves fatigue resistance and low-temperature crack resistance; reduces salt damage and extends service life	[77]
Construction cost	Approximately 2.12 times conventional asphalt (26.82 vs. 12.65 USD/m^2^, 7.5 cm layer)	Conductive additives + electrode installation (recycled metal fiber case); addition material in old literature approx. 15.89 USD/m^2^ (1968 price)	[208,211]
Maintenance costs	Primarily electricity costs + periodic electrode/circuit inspections; significant lifecycle cost advantages	Eliminate/reduce chemical de-icing agents and mechanical snow removal, minimizing damage to road surfaces and the environment	[77,208]

## Data Availability

This article is a review of the existing literature. No new data were created or analyzed in this study. All data discussed or cited are available from the original publications referenced in the reference list.

## Abbreviations

The following abbreviations are used in this manuscript:

ECAC	electrically conductive asphalt concrete
CNTs	carbon nanotubes
